# Utility of automated CT perfusion software in acute ischemic stroke with large and medium vessel occlusion

**DOI:** 10.1002/acn3.52207

**Published:** 2024-10-07

**Authors:** Rezan Ashayeri Ahmadabad, Kim H. Tran, Yiran Zhang, Mahesh P. Kate, Sachin Mishra, Brian H. Buck, Khurshid A. Khan, Jeremy Rempel, Gregory W. Albers, Ashfaq Shuaib

**Affiliations:** ^1^ Division of Neurology University of Alberta Edmonton Canada; ^2^ Neuroscience and Mental Health Institute, University of Alberta Edmonton Canada; ^3^ Division of Radiology and Diagnostic Imaging University of Alberta Edmonton Canada; ^4^ Stanford Stroke Center Stanford University Palo Alto USA

## Abstract

**Background:**

Early diagnosis of large vessel occlusion (LVO) in acute stroke often requires CT angiography (CTA). Automated CT perfusion (CTP) software, which identifies blood flow abnormalities, enhances LVO diagnosis and patient selection for endovascular thrombectomy (EVT). This study evaluates the sensitivity of automated CTP images in detecting perfusion abnormalities in patients with acute ischemic stroke (AIS) and LVO or medium vessel occlusion (MeVO), compared to CTA.

**Methods:**

We screened acute ischemic stroke patients presenting within 24 h who underwent CT, CTA, and CTP as per institutional protocol. RAPID AI software processed CTP images, while neuroradiologists reviewed CTA for intracranial arterial occlusions. Sensitivity, specificity, and accuracy of automated CTP maps in detecting occlusions were assessed.

**Results:**

Of 790 screened patients, 31 were excluded due to lack of RAPID CTP data or poor‐quality scans, leaving 759 for analysis. The median age was 71 years (IQR: 61–81), with 47% female. Among them, 678 had AIS, and 81 had AIS ruled out. CTA identified arterial occlusion in 562 patients (74%), with corresponding CTP abnormalities in 537 patients (Tmax > 6 sec). In the 197 without occlusion, CTP was negative in 161. Automated CTP maps had a sensitivity of 95.55% (CI 95: 93.50–97.10%), specificity of 81.73% (CI 95: 75.61–86.86%), negative predictive value of 98.22% (CI 95: 97.39–98.79%), positive predictive value of 63.54% (CI 95: 56.46–70.09%), and overall accuracy of 85.18% (CI 95: 82.45–87.64%).

**Conclusions:**

Automated CTP maps demonstrated high sensitivity and negative predictive value for LVOs and MeVOs, suggesting their usefulness as a rapid diagnostic tool, especially in settings without expert neuroradiologists.

## Introduction

Recent guidelines recommend endovascular thrombectomy (EVT) as the standard‐of‐care for most large vessel occlusions (LVOs) with favorable neuroimaging up to 24 h from symptom onset or from when the patient was last known to be well.^1^, ^2^, ^3^, ^4^ LVOs are defined as occlusions of the proximal arteries of the anterior and posterior circulation, that is, the A1 segment of the anterior cerebral artery (ACA), the M1 segment of the middle cerebral artery (MCA), the extracranial and intracranial internal carotid artery (ICA), vertebral artery (VA), basilar artery (BA), and the P1 segment of the posterior cerebral artery (PCA).^5^ Cerebral computed tomography angiography (CTA) and CT perfusion (CTP) are increasingly used to ensure precise identification of arterial occlusion site implicated in acute stroke. However, identifying occlusions on CTA can pose challenges in emergency settings,^6^ with one recent study showing a false negative rate of about 30% for CTA alone to detect intracranial occlusions.^7^

Detection of medium‐size vessel occlusions (MeVOs; M2, M3, A2, and A3) using CTA can be even more difficult due to their small sizes, larger number of vessels, poor resolution, and variable anatomy when compared to large vessels. This may be particularly important during the night when neuroradiological expertise may not be widely available and the risk for errors may increase.^8^ Studies have reported the sensitivity for detecting MeVOs on CTA to be as low as 33%,^9^ and 28% of M2 occlusions can be missed on initial CTA evaluations.^10^ Moreover, in a systematic review of 12 studies, CTA demonstrated significantly lower sensitivity compared to CTP in detecting MeVOs (0.74 vs. 0.89, *P* < 0.01).^11^

Several recent studies have shown an increased sensitivity in detection of arterial occlusion when CTP was added to non‐contrast CT (NCCT) and CTA, thereby decreasing the chances of missing EVT eligible patients.^9^, ^12^, ^13^ CTP has been used as an image processing platform for large clinical studies such as DEFUSE 1, 2, 3^14^, ^15^, ^16^; DAWN^17^; EXTEND^18^; EXTEND‐IA,^19^ and SWIFT PRIME.^20^, ^21^

Prompt identification of LVOs or MeVOs requires expertise that may not be immediately available, especially outside of regular working hours.^17^, ^22^, ^23^ This may be more pronounced in smaller hospitals with limited expertise, lower patient loads, or centers in remote areas with only access to NCCT leading to potential delays in identification and treatment of LVOs.^10^, ^24^ Such delays can lead to worsened outcome and, in some cases, exclude patients from eligibility for EVT.^25^

Guidelines recommend either NCCT/CTA or a combination of NCCT/CTA/CTP as the primary imaging modalities for the evaluation of acute stroke patients. There is a lack of prospective data comparing CTA with CTP for optimal first screening imaging modality in real‐world acute ischemic stroke (AIS) settings for detection of LVOs and MeVOs. In this study, we aimed to evaluate the sensitivity of automated CT based perfusion deficit as a surrogate marker of arterial occlusion in anterior circulation infarcts compared to CTA.

## Methods

### Patient selection

This is a single‐center retrospective cohort study of consecutive patients who underwent multimodal stroke imaging (CT/CTA/CTP) for a suspected acute ischemic stroke at the University of Alberta Hospital, Edmonton, Alberta, Canada, between July 2021 and July 2023. Eligible patients were identified by querying electronic medical records and a Picture Archiving and Communication System. Patients included were those (1) aged 18 years or older, (2) presenting with an acute stroke syndrome within 24 hours of onset or last know well, and (3) underwent multimodal imaging including NCCT, CTA, and CTP as part of the initial evaluation. Exclusion criteria were (1) patients with technically inadequate CTP or CTA scans, such as those compromised by poor contrast bolus or severe motion artifacts, (2) no RAPID post‐processing CTP data available, and (3) chronic intracranial occlusions.

The institutional review board approved the study, granting a waiver of written consent due to its retrospective nature and the anonymization of all patient data. This study was initiated by the investigator and received no external financial support.

### CT acquisition, reconstruction, and postprocessing

The study used a 256‐slice multidetector CT to perform a multimodal stroke CT protocol, including NCCT, CTP, and multiphase CTA. The CTP images were acquired during a bolus injection of iodinated contrast material and reconstructed at a 10‐mm slice thickness. The CTP images were processed using a commercially available software platform (RAPID 4.9) to derive the following perfusion maps: Tmax, cerebral blood volume, cerebral blood flow, and mean transit time. Axial CTA images were reconstructed at 1 mm slice thickness. The study also obtained axial, coronal, and sagittal 3 mm thick multiplanar reconstructions and 24 mm thickness maximum intensity projections. Processing of all imaging data was done as part of the clinical standard workup for the patient.

### LVO definition

LVO was defined as an arterial occlusion involving the extracranial ICA, intracranial ICA, M1 segment of the MCA, A1 segment of the ACA, extracranial VA, intracranial VA, BA, and P1 segment of the PCA.^4^ The classification of proximal M2 occlusions is challenging due to anatomical variability in the MCA and the complexity of its branching patterns.^3^ While some institutions consider these M2 occlusions as large or proximal vessel occlusions, they are not considered as such by American Heart Association guidelines.^1^ However, the role of EVT for M2 occlusions is the focus of ongoing randomized trials, and they are more difficult to detect on CTA compared to M1 occlusions.^1^, ^3^ Due to these challenges, they are included in MeVO subcategory in this study.

### MeVO definition

MeVO was defined as an arterial occlusion involving M2, M3, A2, A3, P2, and P3 which was visible on the initial CTA and was reported by a neuroradiologist.^26^

### Reference standard

CTAs and CTPs of all consecutive patients included in the study underwent thorough review. The reference standard for the presence of LVO or MeVO was defined based on neuroradiologist reporting of the CTA. Automated CTP maps in detecting occlusions. For CTP abnormalities, automated CTPs maps were evaluated. An abnormal CTP was defined as any volume of tissue with a T‐max abnormality over 6 seconds.

### Statistical analysis

SPSS Version 29.0.1.1 was used for data management and descriptive statistical analysis. Median and interquartile range (IQR) were calculated to summarize the age distribution within the study population. Frequency counts and percentages were computed for categorical variables such as sex (male and female) to describe their distribution across the entire sample and within different subgroups. Cross‐tabulations were conducted to explore associations or patterns among dichotomous variables of interest.

The calculation of diagnostic test parameters, including sensitivity, specificity, accuracy, positive predictive value (PPV), negative predictive value (NPV), and receiver operating characteristic (ROC) curves was performed using MedCalc® Statistical Software version 22.030 (MedCalc Software Ltd, Ostend, Belgium; https://www.medcalc.org; 2024). We employed ROC curve methodology to evaluate the diagnostic performance of three Tmax abnormalities: >4, >6, and >8 sec. These thresholds were assessed for their ability to discriminate between patients with arterial occlusion and those without. ROC curves were constructed to visualize the sensitivity and specificity of each Tmax threshold in predicting arterial occlusion. Area under the ROC curve (AUC) values were calculated to quantify the overall discriminatory power of each threshold.

## Results

**Figure 1:**
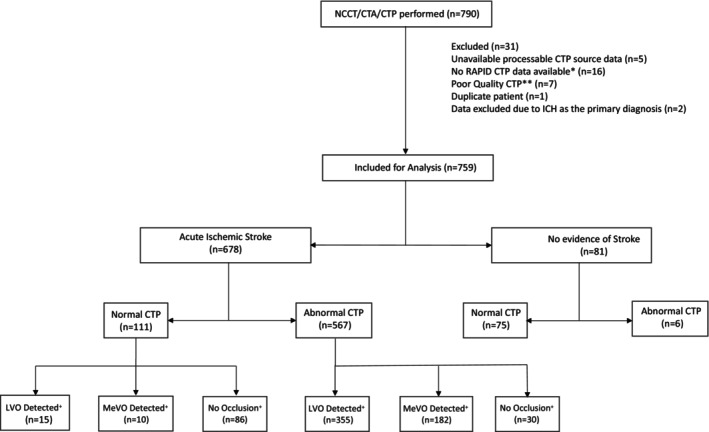
Flowchart of patient selection. *Rapid CTP is an automated software with an FDA indication to aid in the selection of patients for acute stroke therapy. **Poor‐quality CTP results included significant patient motion, extensive curve truncation, delayed contrast arrival or improper timing in CTP, or notable artifacts present in the CTP source data. + Occlusion detected by CTA report. CTA, computed tomography angiography; CTP, computed tomography perfusion; DSA, digital subtraction angiography; ICH, intracerebral hemorrhage; LVO, large vessel occlusion; NCCT, non‐contrast computed tomography; MCA, middle cerebral artery; MeVO, medium vessel occlusion; PCA, posterior cerebral artery.

The study enrolled 790 patients, of whom 31 were subsequently excluded, resulting in a final cohort of 759 patients for the analysis. The common reasons for patient exclusion included unavailable processable CTP source data (*n* = 5), lack of RAPID CTP data (*n* = 16), and instances of poor‐quality CTP scans (*n* = 7). Additionally, one patient was excluded due to duplicate records, and two patients were excluded due to a primary diagnosis of intracerebral hemorrhage (ICH). The patient selection process is illustrated in a flowchart (Fig. 1).

Of the 759 patients included in our analysis, median age of the study population was 71 years (interquartile range: 61–81), with 47% being female. The majority (*n* = 678) had a final diagnosis of AIS, while acute stroke was not identified in the remaining (*n* = 81) patients.

Among the 678 patients with a final diagnosis of ischemic stroke, 111 showed normal results on CTP. Of these, 25 patients had arterial occlusions (15 cases of LVO and 10 cases of MeVO), where RAPID imaging failed to detect abnormalities in Tmax over 6 sec. The remaining patients did not present with any intracranial occlusions.

The remaining 81 patients were diagnosed with conditions other than AIS. Of these patients 75 had normal CTP results, while 6 had Tmax > 6 sec abnormality. None of these patients showed acute arterial occlusion on CTA.

In our primary analysis of the 759 included patients, we adopted any volume of 1 mL or more of hypoperfused tissue on the Tmax > 6 sec map as the definition for CTP abnormality, aligning with the processing protocol in RAPID software. The automated CTP maps showed a sensitivity of 95.55% (CI 95: 93.50–97.10%), specificity of 81.73% (CI 95: 75.61–86.86%), negative predictive value of 98.22% (CI 95: 97.39–98.79%), positive predictive value of 63.54% (CI 95: 56.46–70.09%), and overall accuracy of 85.18% (CI 95: 82.45–87.64%).

**Figure 2:**
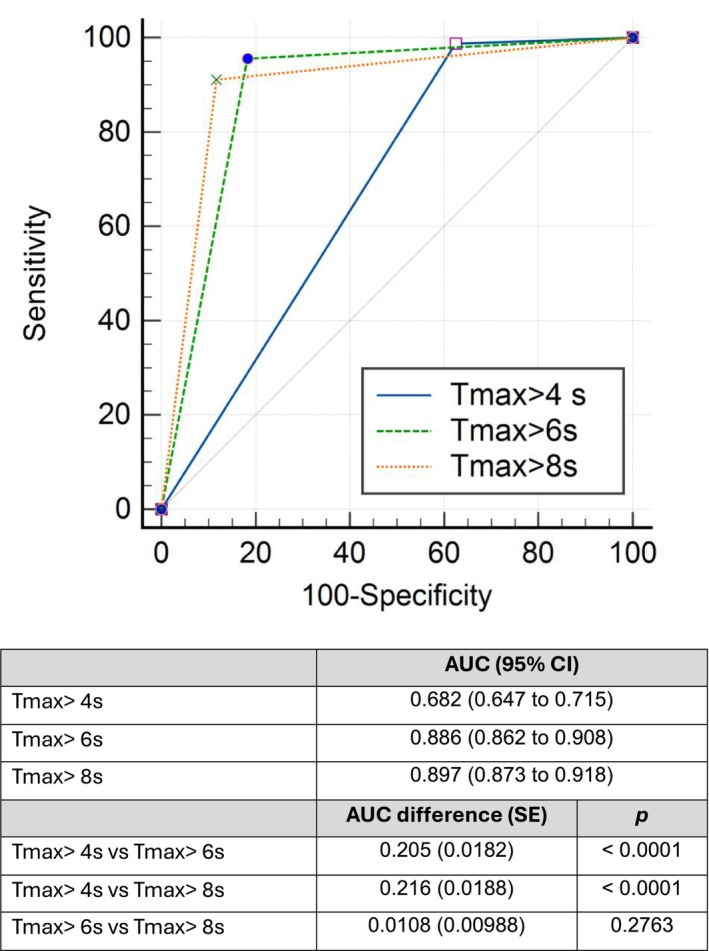
ROC curves to assess the sensitivity and specificity of CTP abnormalities at three different Tmax thresholds (Tmax > 4 sec, Tmax > 6 sec, and Tmax > 8 sec) in distinguishing between arterial occlusion and no occlusion. AUC, area under the curve; CI, confidence interval; CTP, CT perfusion; Tmax, time to maximum contrast; LVO, large vessel occlusion; MeVO, medium vessel occlusion; ROC, receiver operating characteristic; SE, standard error. AUC comparison was performed with DeLong test. Outcome of interest was presence or absence of LVO or MeVo.

The diagnostic performance of CTP abnormalities at three different Tmax thresholds (Tmax > 4 sec, Tmax > 6 sec, and Tmax > 8 sec) in distinguishing between occlusion and no occlusion was also assessed as a secondary analysis using ROC curves in Figure 2. The AUCs indicate varying discriminatory abilities. Comparative analysis reveals significant differences between Tmax >4 seconds and the other thresholds, while Tmax > 6 sec and Tmax > 8 sec show no significant difference in their predictive capabilities (*P* = 0.2763).

**Figure 3:**
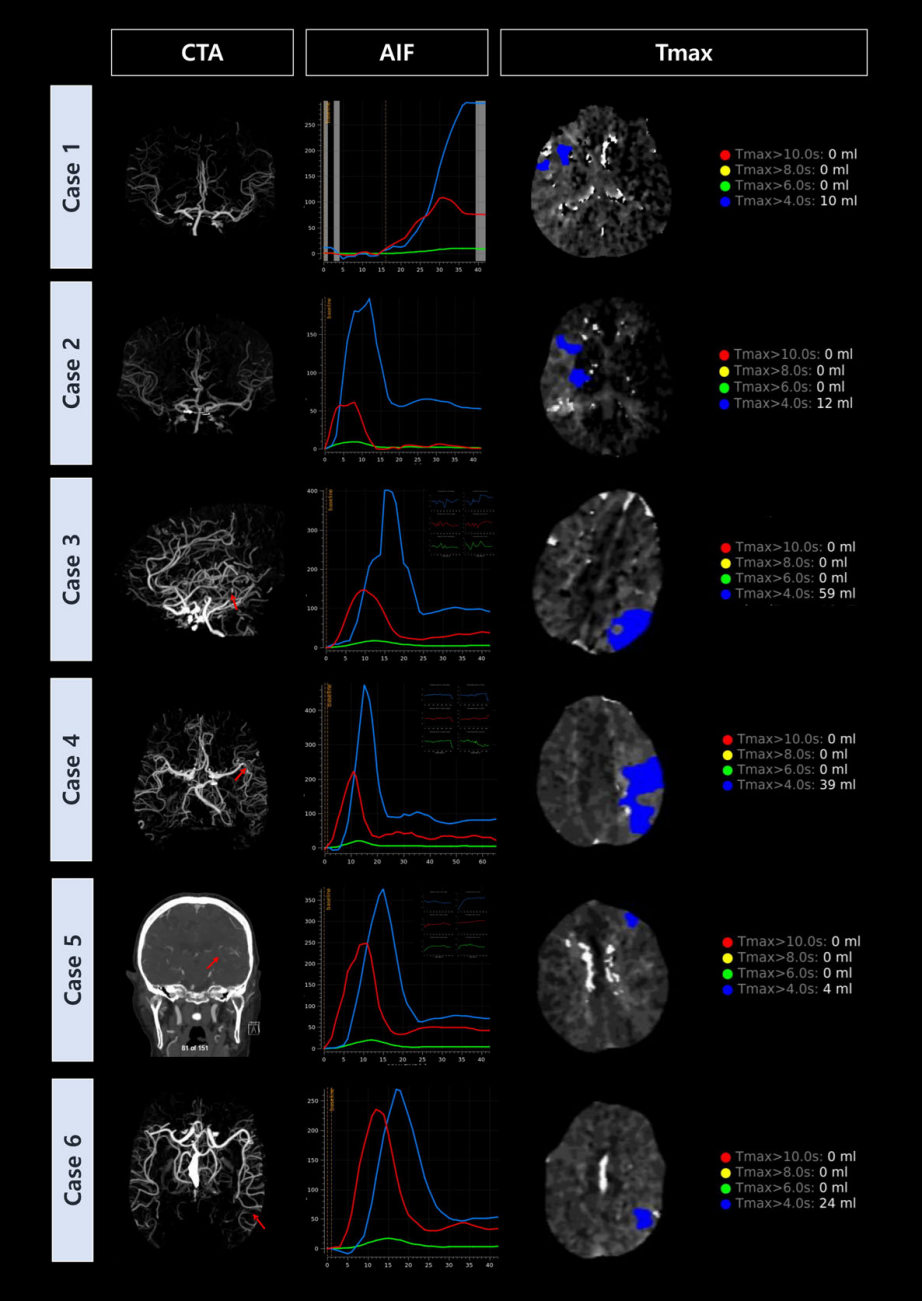
Six patients who did not demonstrate concordance between CTP and CTA. In all patients, if abnormality for Tmax threshold is set above 4 sec, perfusion deficit could be detected. Case 1: Right MCA‐M1 occlusion. The early truncation of AIF and VOF curves limited the accurate estimation of Tmax, contributing to the mismatch threshold fallacy. Case 2: Right MCA‐M1 occlusion. Insufficient time for baseline CT attenuation affected the precise estimation of Tmax, highlighting the importance of a 5–10 sec pre‐contrast baseline (indicated by two parallel dash lines before the slope) for optimal scans. Case 3: Left MCA‐M2 occlusion. Inadequate baseline CT attenuation and motion artifact during scanning compromised the accurate estimation of Tmax, contributing to the threshold fallacy observed. Case 4: Left MCA‐M2 occlusion. Similar challenges with baseline CT attenuation and motion artifact affected the reliability of Tmax estimation, influencing the discordance between CTP and CTA findings. Case 5: Left distal MCA‐M3 occlusion. Challenges with baseline CT attenuation and motion artifact during the scan compromised the accurate estimation of Tmax, contributing to the observed threshold fallacy. Case 6: Left distal MCA‐M3 occlusion. Insufficient baseline CT attenuation affected the precise estimation of Tmax, highlighting another instance of the threshold fallacy in CTP imaging. CTP, CT perfusion; CTA, CT angiography; Tmax, maximum contrast transit time; MCA, middle cerebral artery; AIF, arterial input function; VOF, venous output function.

We randomly reviewed six patients who were false‐negatives (Fig. 3): two with LVO and four with MeVO in the intracranial anterior circulation, all of whom had normal findings on CTP imaging. Both LVO patients had atrial fibrillation (AF), which can impact cerebral perfusion. One patient faced technical challenges during CTP imaging. Notably, CTA revealed adequate collaterals in these cases, potentially explaining the discrepancy between CTP and CTA results. Both LVO cases underwent EVT. Among the four patients with MeVOs, one had a focal occlusion in the M2 segment, another presented with proximal M2 involvement, while two cases were diagnosed with distal M3 segment involvement, including one suspected distal M3 occlusion. In one MeVO case, EVT was performed due to a nonocclusive thrombus in a chronically stenotic artery. AF was present in one of the MeVO cases.

## Discussion

In this retrospective cohort study, we found that automated CTP accurately detects perfusion deficits associated with anterior circulation LVO and MeVO infarcts, assisting in timely and precise clinical decision‐making. Our analysis focused on measuring the sensitivity of automated CTP maps. We particularly focused on patients with normal CTP results who had signs of LVO or MeVO on their CTA scans. The automated CTP maps showed high sensitivity (95.55%) and negative predictive value (98.22%).

If CTP alone were used for screening, we may have missed 25 out of 562 (4.44%) arterial occlusions (15 LVOs and 10 MeVO). This rate is lower compared to relying solely on CTAs. A study by Duvekot et al^27^ found that local radiologists interpreting CTA alone, missed a higher proportion of LVOs (7%) and MeVOs (38%). However, their study reported a high specificity for CTA in detecting LVOs (100%) and MeVOs (99%), contrasting with our findings of CTP specificity at 81.7%. Our study primarily focused on treatable LVOs and MeVOs potentially overlooked by CTP, highlighting the sensitivity and negative predictive value of automated CTP.

The diagnostic performance of CTP abnormalities at three Tmax thresholds (Tmax > 4 sec, Tmax > 6 sec, and Tmax > 8 sec) was assessed using ROC curves. A Tmax > 6 sec threshold yielded the highest diagnostic accuracy with an AUC of 0.905 (SE = 0.015), indicating it is the most effective in distinguishing between occlusion and no occlusion. The Tmax > 4 sec threshold had an AUC of 0.869 (SE = 0.019), and the Tmax > 8 sec threshold had an AUC of 0.878 (SE = 0.016), both demonstrating good diagnostic accuracy but lower than the Tmax > 6 sec threshold.

MeVO‐related infarctions are common and can lead to large disabling strokes.^28^ EVT is increasingly being recognized as a treatment option in most cases secondary to MeVO. Currently, ongoing clinical trials upon completion will, we hope, allow for better understanding of EVT in the management of MeVO strokes.^28^ The challenges of rapid diagnosis of MeVOs are compounded when identifying MeVOs due to their smaller size, variable anatomy, and lower detection rates on initial CTA evaluations.^8^, ^9^, ^10^

A recent study by Fasen et al^10^ showed that single‐phase CT angiography misses M2 occlusions in up to 35% of cases, underscoring the potential of automated CTP to intervene in EVT opportunities. Another study^8^ compared the accuracy of territorial Tmax delay with CTA in identifying distal MeVOs, and found Tmax delay had a sensitivity of 100% and specificity of 87.8% for detecting distal medium vessel occlusions (DMVOs), with diagnostic accuracy and efficiency considerably higher than CTA (*P* < 0.001).

The clinically relevant Tmax thresholds in CT perfusion commonly include Tmax > 4 sec, Tmax > 6 sec, and Tmax > 8 sec, with Tmax > 6 sec often indicating the presence of penumbra.^29^ In our study, we defined a CT perfusion abnormality using a threshold of Tmax > 6 sec, considering values below this threshold as normal. Interestingly, among cases interpreted as normal on CTP, both LVO cases and three out of four MeVO cases had genuine, albeit milder, Tmax delays ranging between 4 and 6 sec, as depicted in Figure 3. This observation prompts reconsideration of Tmax delay thresholds for defining penumbra. It is important to emphasize that reliance solely on core/penumbra maps can be misleading. Our findings highlight the necessity of examining individual perfusion maps, such as the Tmax map, which can provide critical insights not evident from core/penumbra maps alone. The Tmax map often reveals sub‐threshold perfusion lesions that might otherwise be missed.

Our findings are consistent with prior studies, where 90% of DMVOs were identified using a Tmax threshold of 6 sec delay, while the remaining 10% showed milder delays between 4 and 6 sec.^8^ Recent hypotheses suggest that DMVOs may face less blood flow delay compared to proximal LVOs due to shorter collateral routes.^3^

Our study aimed to address these challenges by evaluating the sensitivity of automated CT‐based perfusion deficits as a surrogate marker for arterial occlusion in anterior circulation infarcts compared to CTA. Our findings showed high sensitivity, reasonable specificity, and diagnostic accuracy of automated CTP maps in detecting territorial infarctions associated with both LVOs and MeVOs, consistent with previous trials and reviews.^14^, ^15^, ^16^, ^17^, ^18^, ^19^, ^20^, ^21^ This capability offers valuable insights for clinicians, particularly in settings with limited access to experienced neuroradiologists, facilitating expedited decision‐making regarding patient triage and treatment.

The discrepancies between the results of CTP and CTA in a subgroup of patients underscore the complexity of stroke imaging interpretation, particularly in cases involving technical challenges or patients with conditions such as atrial fibrillation (AF), heart failure, or carotid stenosis, which can result in compromised cerebral perfusion.^30^ Reduced cardiac output in heart failure and irregular heartbeats associated with AF can result in systemic hypoperfusion, including decreased cerebral blood flow. In CTP imaging, this can result in variations in contrast agent arrival time, bolus shape, and perfusion parameters mainly described as low‐attenuation arterial input function (AIF) curve with slow upstroke and broad peak. Significant carotid stenosis can also impair cerebral blood flow by reducing the supply of oxygenated blood to the brain. In CTP imaging, this can lead to regional hypoperfusion or delayed arrival of contrast agent in affected brain territories. This can result in perfusion deficits similar to heart failure and AF or artifacts in CTP images, impacting the interpretation of cerebral perfusion status. Overall, AF, heart failure, and carotid stenosis can all influence cerebral perfusion and potentially compromise CTP results by altering cardiac function, systemic hemodynamics, and regional blood flow dynamics in the brain.^30^ Understanding these physiological effects is essential for accurate interpretation of CTP imaging findings in patients with these conditions.

Table 1 summarizes the common pitfalls in CT perfusion studies which can be classified into technical and clinical factors. Technical factors includes issues such as misplaced AIF or venous output function (VOF) markers due to the patient motion, which can result in abnormal perfusion maps.^30^ This may be reduced by training staff and educating patients, though it can sometimes be challenging in acute settings. The position of AIF markers can also affect map accuracy, with placement distal to the occlusion site potentially leading to misinterpretation.^31^ Other technical factors including slow contrast delivery may also affect map quality, which should be taken into account for getting interpretable maps.^32^, ^33^

**Table 1:** Common pitfalls in CT perfusion (CTP) analysis in acute ischemic stroke.

Technical factors	
Patient motion	Leading to misplaced AIF or VOF, resulting in an irregular or fluctuating pattern referred to as a jagged, multi‐peak arterial input function^30^
AIF distal to an occlusion	May not be reflected on the time attenuation curve, requires close attention to AIF placement^31^
Slow injection rate	Low‐attenuation AIF curve (with slow upstroke and broad peak) (<100 HU)^32^, ^33^
Slow or absent intravenous saline chase	
Artifacts in skull base, posterior fossa, or orbits	Poor attenuation curves, overestimation of penumbra^33^
Clinical factors	
Poor cardiac output	Low‐attenuation AIF curve (with slow upstroke and broad peak) (<100 HU), overestimation of penumbra^32^, ^33^
Cardiac arrhythmia	
Proximal arterial stenosis or occlusion	
Presence of good collaterals	Underestimation of the core infarct, especially in the setting of reperfusion therapy or in strokes with extended time windows^34^
Hyperacute strokes who received reperfusion therapy	Overestimation of the core infarct, “Ghost infarct” phenomenon^35^
Stroke mimics, especially seizure	Hyperperfusion in peri‐ictal phase and hypoperfusion in post‐ictal phase^36^

AIF, arterial input function; HU, Hounsfield unit; VOF, venous output function.

Clinical pitfalls include underestimation and overestimation of core infarct and penumbra. Automated software may underestimate core infarct size, particularly in cases with extensive leptomeningeal or pial collaterals.^34^ Conversely, complete reperfusion therapy can lead to core infarct overestimation.^35^ Misclassification of penumbra is common and can be caused by artifacts or conditions affecting arterial flow. Stroke mimics, especially seizures, further complicate interpretation.^36^ It leads to hyperperfusion in the peri‐ictal phase and hypoperfusion in the post‐ictal phase, which can be quite easily identified on CTP maps, particularly in the context of the clinical scenario. These variations highlight the importance of a multimodal imaging approach and the need for refining imaging protocols to optimize diagnostic accuracy in AIS settings.

We now underscore that CTP should be interpreted as part of a multimodal imaging approach. A comprehensive evaluation must include NCCT, CTA, and CTP maps. This integrated approach ensures a more accurate assessment of perfusion and directs attention to areas that require detailed examination.

## Limitations

Our study has several limitations that should be acknowledged. Firstly, the retrospective nature and reliance on electronic medical records and imaging databases may introduce selection bias. Secondly, the single‐center design may limit the generalizability of our findings. Thirdly, our results are solely based on neuroradiologists' reports, which may lead to improved interpretation of CTA when CTP imaging is available before the report release. The accuracy of reporting LVOs and MeVOs when only CTA was performed remains uncertain. Additionally, the high proportion of patients with poor‐quality studies that were not interpretable (*n* = 12, 1.5%) is also a concern.

## Conclusion

In our study, automated CTP maps showed high sensitivity, negative predictive value, specificity, and accuracy in patients with LVOs and MeVOs. Our findings suggest that automated CTP maps serve as valuable rapid diagnostic screening tools, especially in settings where expert neuroradiologists may not be readily available. The use of automated CT perfusion studies with RAPID can effectively rule out candidates for thrombectomy, provided they are conducted consistently and interpreted accurately.

Further prospective studies are needed to validate our findings and evaluate how automated CTP impacts clinical outcomes and treatment decisions in real‐world practice.

## Author Contributions

RAA assisted with data collection, drafting the main manuscript, writing, and editing. KHT and YZ were involved in data collection and editing, while MPK and BHB contributed to research design, conception, and statistical analysis. SM helped with writing and editing, and KAK, JR, and GWA contributed to research design and conception. AS was involved in research design and conception, as well as writing and editing.

## Conflict of interest

Gregory W. Albers is a consultant for Genentech and iSchemaView and holds equity in iSchemaView. The other authors have no conflicts of interest.

## Data Availability

The data that support the findings of this study are available from the corresponding author upon reasonable request.
